# Diagnostic odyssey of a German shepherd dog with disseminated *Penicillium labradoris* infection: a case report

**DOI:** 10.3389/fvets.2026.1611862

**Published:** 2026-03-06

**Authors:** Marta Medardo, Paolo Capozza, Mara Miglianti, Alessio Xenoulis, Giulio Cocciolo, Michele Marino, Paola Rigamonti, Gino Pinotti, Piera Anna Martino, Claudia Cafarchia, Vito Martella, Nicola Decaro

**Affiliations:** 1 Veterinary Analysis Laboratory MyLav La Vallonea, Milan, Italy; 2 Department of Veterinary Medicine, University of Bari “Aldo Moro”, Valenzano, Bari, Italy; 3 Orobica Veterinary Clinic, Bergamo, Italy; 4 One Health Unit, Department of Biomedical, Surgical and Dental Sciences, University of Milan, Milan, Italy; 5 Department of Pharmacology and Toxicology, University of Veterinary Medicine, Budapest, Hungary

**Keywords:** canine osteomyelitis, disseminated fungal infection, *Penicillium labradoris*, opportunistic mycosis, diagnostic challenge

## Abstract

Below, the first confirmed canine infection caused by *Penicillium labradorum* (syn. *P. labradoris*) in Italy is reported, diagnosed using a multidisciplinary approach. In November 2021, a 10-year-old spayed female German Shepherd dog with urinary incontinence was presented. Physical examination of the right forelimb revealed a non-painful soft tissue enlargement with firm consistency, while peripheral lymph nodes were unremarkable. Hematological, serum biochemical, and urinalysis tests showed no significant abnormalities. Radiographic examination revealed a proliferative lesion of the right radial bone, whereas abdominal ultrasonography did not identify anatomical alterations. Computed tomography (CT) revealed aggressive proliferative and lytic lesions of the radius associated with soft tissue oedema. Cytological examination of fine-needle aspirates showed marked neutrophilic and moderate macrophagic inflammation with evidence of fungal hyphae. Histological examination of a bone biopsy revealed severe fibroplasia and fibrosis associated with mixed inflammatory infiltrates, and Grocott-Gomori's methenamine silver (GMS), and Periodic acid-Schiff (PAS) staining confirmed the presence of fungal hyphae. Bone biopsy and urine cultures yielded fungal growth, which was morphologically identified as *Penicillium* spp. Molecular identification, based on amplification and sequencing of the nuclear ITS region, β-tubulin, and calmodulin genes from urine and biopsy samples, confirmed the isolate as *Penicillium labradorum*. The dog was treated with itraconazole (11 mg/kg). Approximately 190 days after the initial diagnosis, the dog died; necropsy was not performed because owner consent was not granted. Disseminated fungal infections are often associated with a poor prognosis due to delayed diagnosis. This case highlights that fungal infections should always be considered in the differential diagnosis of bone lesions in dogs.

## 1 Introduction

Disseminated fungal infections in dogs are uncommon but often fatal, largely due to difficulties in achieving timely diagnosis. In dogs, disseminated mold infections are most frequently associated with *Aspergillus* spp., followed by other filamentous fungi such as *Scedosporium, Fusarium*, and less frequently *Penicillium* species, which are increasingly recognized as true opportunistic pathogens rather than mere environmental contaminants (1–3). When bone lesions are identified, fungal etiologies should always be considered in the differential diagnosis (1). Here, we report the first confirmed case of *Penicillium labradorum* (syn. *P. labradoris*) infection in a dog in Italy, diagnosed through a multidisciplinary approach integrating imaging, cytology, culture, and molecular identification. This case expands the known geographical distribution of *P. labradorum* and contributes to the limited body of knowledge regarding invasive *Penicillium* infections in canine hosts. Although *Penicillium* spp. are commonly regarded as environmental molds or laboratory contaminants, several species have increasingly been recognized as true pathogens in both humans and animals, particularly dogs (1). According to the most recent taxonomic revision of the genus *Penicillium, P. labradoris* belongs to the subgenus *Aspergilloides*, section *Exilicaulis*, series *Erubescentia* (4). Species within section *Exilicaulis* are characterized by mono- and bi-verticillate conidiophores and non-vesiculate stipes. Notably, two species from this section have been reported as causative agents of osteomyelitis (*P. canis*) and disseminated infection (*P. labradoris*) in dogs (3, 5, 6), as well as invasive pulmonary infection, fungemia, paravertebral infection, and mycetoma in humans (*P. decumbens*) (7). *Penicillium* spp. are capable of invading a wide range of organs, including the lungs, liver, musculoskeletal system, lymphatic system, central nervous system, and kidneys, and may disseminate systemically in both canine and human hosts. Since 1990, a total of 22 canine cases of infection caused by *Penicillium* and *Talaromyces* species have been reported. Among these cases, 36% (8/22) were associated with osteomyelitis, 27% (6/22) with disseminated infection, 14% (3/22) with pneumonia, 9% (2/22) with encephalitis, and 14% (3/22) with other clinical presentations, including lymphangitis, dermatitis, and abdominal abscesses. Within this group, only a limited number of cases have been specifically attributed to *Penicillium* species, highlighting the rarity of confirmed canine penicilliosis and the clinical relevance of the present report (3). Overall prognosis has been poor, particularly in cases of disseminated infection, with limited response to antifungal therapy alone. Favorable outcomes have been reported mainly in localized infections. Treatment regimens have included various azole antifungals (e.g., ketoconazole, itraconazole, and voriconazole), sometimes combined with terbinafine or amphotericin B, and occasionally surgical intervention. *In vitro* studies have shown favorable activity of terbinafine and echinocandins against *Penicillium* spp., whereas amphotericin B and azoles demonstrate variable efficacy (3, 8, 9). In humans, invasive *Penicillium* infections are most commonly associated with immunosuppression, including corticosteroid therapy, chemotherapy, or underlying immunodeficiency. Approximately 30 cases have been reported in immunocompromised patients since the 1990s (1, 7, 10). In dogs, additional factors such as climatic conditions and breed-related immune defects have been proposed. German Shepherd Dogs—particularly young females—and Labrador Retrievers appear predisposed to systemic fungal infections, possibly due to inherited immunological defects associated with reduced IgA concentrations (3, 11). These breeds account for approximately 41% (9/22) of reported canine *Penicillium* infections (3). However, a recent review highlighted that most affected dogs (90%, 20/22) lacked identifiable predisposing factors (3).

In this case report, we describe a disseminated infection caused by *P. labradoris* in a German Shepherd Dog, aiming to expand current knowledge on invasive fungal infections in companion animals and to support the development of more accurate diagnostic strategies and appropriate clinical management.

## 2 Material and methods

### 2.1 Case description

**Figure 1:**
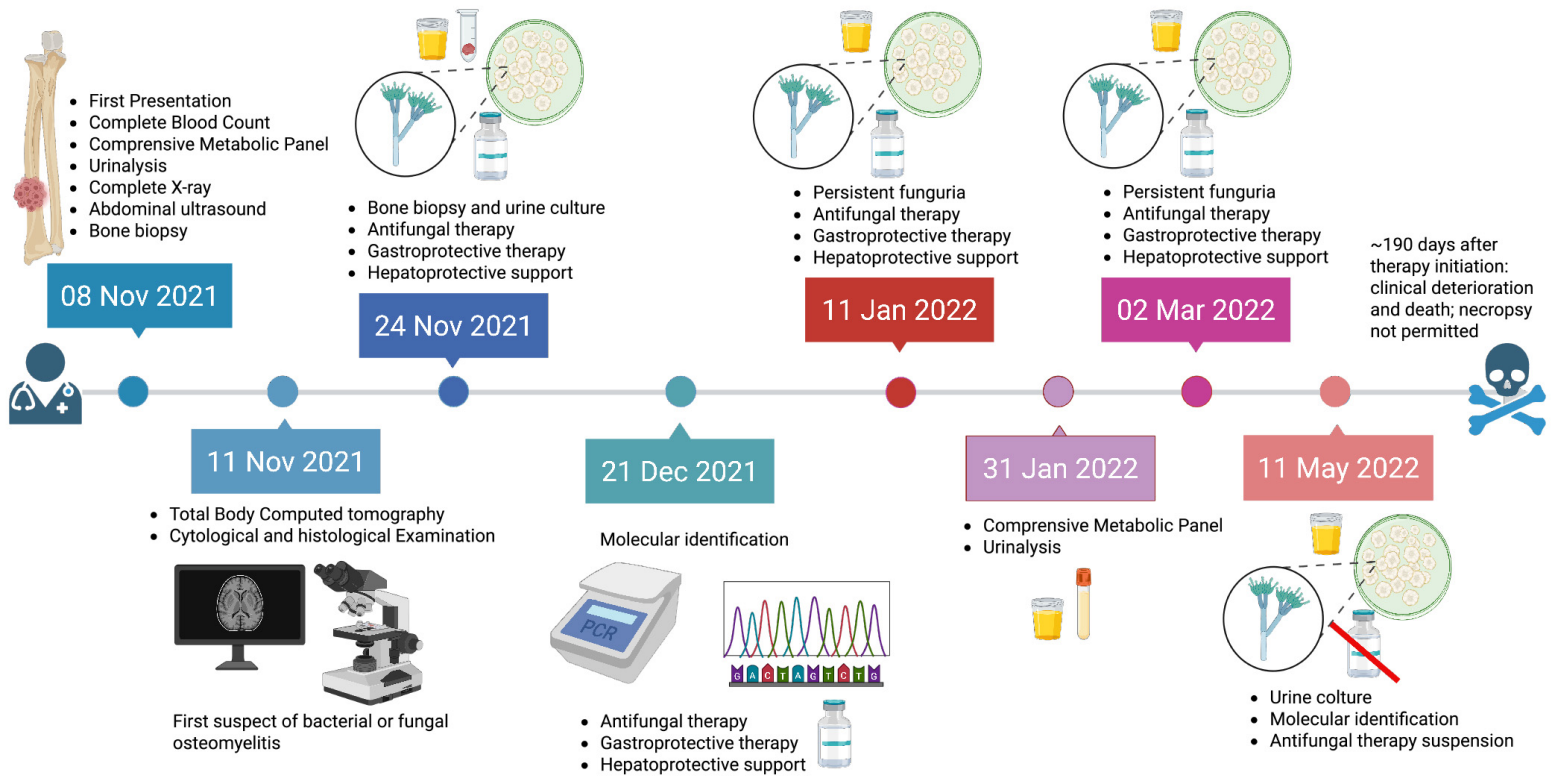
Timeline illustrating the clinical course, diagnostic workflow, microbiological findings, and therapeutic interventions of the case. The figure summarizes the sequence of events from the initial clinical presentation with an incidentally detected bone lesion, through advanced imaging, histopathological examination, fungal culture, and molecular identification of the etiological agent, to antifungal treatment and follow-up. The initiation of itraconazole therapy, the persistence of funguria despite prolonged antifungal treatment, and the subsequent discontinuation of therapy due to microbiological failure are explicitly highlighted. Events occurring in 2021 and 2022 are distinguished by different color codes to indicate the temporal progression of the case. Icons are used to represent the main diagnostic procedures (imaging, microscopy, PCR and sequencing), microbiological findings (urine culture), and therapeutic steps (antifungal treatment). The skull symbol denotes the unfavorable clinical outcome of the disease.

In November 2021, a 10-year-old female neutered German Shepherd dog, regularly vaccinated and with no known previous systemic diseases, was presented to a private veterinary practice in the Province of Bergamo (Lombardy region, Northern Italy) for clinical evaluation in the context of ongoing phenylpropanolamine (Propalin^®^) therapy (1 mg/kg) for urinary incontinence. The dog was owned, kept under controlled conditions, and had access to the outdoor environment. Clinical examination revealed a firm, non-painful swelling of the right forelimb in the right front paw. The peripheral lymph nodes were palpably normal. Hematological, serum biochemical, and urinalysis tests as well as a complete X-ray of lateral-lateral thorax and lateral-medial right radio projections, ultrasound examinations of the abdomen, and total body computerized tomography (CT) were performed. Jamshidi needle biopsy samples from the osseous lesions and cystocentesis urine were collected for histological and microbiological investigations. All the collected samples were sent to Veterinary Analysis Laboratory MyLav La Vallonea (Milan, Italy) for microbiological examination. Based on microbiological and histopathological findings, antifungal therapy was initiated with itraconazole (11 mg/kg, orally, once daily). Pantoprazole (1 mg/kg, orally, once daily) was administered as gastrointestinal protection. Given the need for prolonged azole therapy, hepatic support with Glutamax^®^ and Besame^®^ was concurrently prescribed as hepatoprotective supplementation, administered according to the manufacturer's instructions. Itraconazole was selected as first-line therapy for suspected systemic fungal infection, in accordance with standard clinical practice, despite the fact that definitive identification of the causative pathogen was not yet available at the time treatment was initiated. Therapeutic follow-up was performed by collecting urine samples every two months. Approximately 190 days after initiation of therapy, the clinical condition worsened, and the dog died. Owner consent for necropsy was not granted. The chronological sequence of clinical procedures, diagnostic investigations, microbiological analyses, and therapeutic interventions performed during the case is summarized in Figure 1.

### 2.2 Complete blood count, biochemical tests, and urinalysis

A comprehensive blood panel was conducted to assess the dog's overall health and to monitor any signs of infection or inflammation. This included a complete blood count (CBC) and the biochemical tests, including the comprehensive metabolic panel (CMP). Urinalysis was conducted to assess kidney function and detect any signs of infection. This included urine chemistry and physical analysis to evaluate color, appearance, specific gravity, pH, and the presence of proteins, glucose, urobilinogen, bilirubin, ketones, erythrocytes, and hemoglobin. Additionally, urine sediment analysis was performed to identify leukocytes, erythrocytes, casts, epithelial cells, crystals, and bacteria.

### 2.3 Diagnostic imaging

Preliminary conventional radiography of the right forelimb and thorax was performed using multiple projections (craniocaudal, mediolateral, oblique) to optimally visualize the anatomical structures of interest. The examination allowed for an assessment of the bones, joints, soft tissues, and any morphological or densitometric alterations. A high-frequency linear transducer was employed to perform a comprehensive abdominal ultrasound examination, utilizing a subcostal and suprapubic acoustic window. Multiple imaging planes were acquired during both the filling and emptying phases of the urinary bladder to provide a detailed assessment of the morphology and function of the lower urinary tract organs. Subsequently, to evaluate urinary incontinence and a recently detected lump on the right forelimb, the patient received a thoracic, abdominal, and forelimb computed tomography (CT) scan at the Orobica Veterinary Clinic.

### 2.4 Cytological and histopathological examination

Tissue samples were obtained from the target lesions using a Jamshidi needle biopsy and subjected to histological and microbiological analysis. Urine samples were collected via cystocentesis and analyzed through microbiological culture and cytological evaluation of the urinary sediment. Biopsy tissue and centrifuged urine sediment were air-dried and subjected to Romanowsky staining (May-Grünwald-Giemsa, Merck KGaA, Darmstadt, Germany) for cytological analysis. Biopsy specimens for histopathological analysis were processed utilizing standard histological techniques, which encompassed fixation in 10% buffered formalin, dehydration via a graded alcohol series, clearing with xylene, paraffin embedding, microtomy at 4 μm, and staining with hematoxylin and eosin (H&E), Grocott-Gomori's methenamine silver (GMS), and Periodic acid-Schiff (PAS) stains (12).

### 2.5 Microbiological investigations

Tissue and urine sediment specimens were inoculated onto selective and differential culture media, including Sabouraud Dextrose Agar with Chloramphenicol (SABC), Columbia Sheep Blood Agar, and Brilliance UTI Clarity Agar (Thermo Fisher Scientific-Oxoid, UK). The inoculated plates were incubated under aerobic conditions at 37°C for a period of seven days. Fungal colonies that developed were subjected to a meticulous phenotypic characterization, including macroscopic and microscopic examination, to determine their genus-level identification (13).

### 2.6 Molecular identification

Molecular identification of fungal isolates was accomplished through a polyphasic approach involving PCR amplification and Sanger sequencing of the β-tubulin, calmodulin, and nuclear ITS regions. Briefly, DNA extraction from all pure microbial cultures collected and from biopsy samples was carried out by automated DNA extractor (QIAsymphony SP instrument, Qiagen, Milan, Italy), following the manufacturer's instructions. The obtained DNA extracts were processed by PCR to amplify the three different genes, including β-tubulin, calmodulin, and nuclear ITS region (Table 1).

**Table 1:** List of oligonucleotides used in this study.

**Primer pair**	**Target zone**	**Sequence (5^′^–3^′^)**	**References**
Bt2a	β-tubulin gene	GGTAACCAAATCGGTGCTGCTTTC	(14)
Bt2b		ACCCTCAGTGTAGTGACCCTTGGC	
Cf1	Calmodulin gene	GCCGACTCTTTGACYGARGAR	(15)
Cf4		TTTYTGCATCATRAGYTGGAC	
I5	ITS and partial lsu rDNA	GGAAGTAAAAGTCGTAACAAGG	(15, 16)
D2r		TTGGTCCGTGTTTCAAGACG	

The PCR was done in a final reaction volume of 25 μL containing 12.5 μL of 2x Platinum^*TM*^ SuperFi^*TM*^ PCR Master Mix (Invitrogen^*TM*^, Milan, Italy), 0.3 μM of each primer, and 2 μL of DNA template; the reaction was brought to the final volume with PCR water. The PCR products were separated by capillary gel electrophoresis using the QIAxcel Advanced (Qiagen, Milan, Italy) and represented as electropherograms by the QIAxcel ScreenGel Software, version 1.5 (Qiagen). Direct sequencing was performed on the PCR products using the BigDye^*TM*^ Terminator v3.1 Cycle Sequencing Kit (Applied Biosystems^*TM*^, Milan, Italy) and a SeqStudio^*TM*^ 8 Flex Genetic Analyzer (Applied Biosystems^*TM*^, Milan, Italy). The acquired sequences were analyzed using the Sequencing Analysis Software v7.0 (Applied Biosystems^*TM*^, Milan, Italy). The sequences were used for the taxonomic recognition of the fungal isolate by BLASTn search of similar sequences deposited in the GenBank databases. These reference sequences included both clinical and environmental isolates of *Penicillium* spp., some of which were previously reported in canine and human infections. All accession numbers are provided in Supplementary Figures S9–S11, allowing full traceability of the comparative data. Evolutionary analyses were conducted in MEGA11 (17–19).

## 3 Results

### 3.1 Complete blood count, biochemical tests, and urinalysis

Hematological analysis revealed a complete blood count (CBC) within reference intervals, except for a mild leukocytosis characterized by a relative monocytosis. Biochemical profiling demonstrated serum concentrations of electrolytes, renal function markers, and hepatic enzymes within normal reference ranges (20, 21). A slight elevation in total serum protein was noted. Urinalysis indicated a specific gravity below the reference range (1015) but was otherwise unremarkable (Table 2). Microscopic examination of the urine sediment revealed no significant cellular casts or crystals, and the quantitative analysis of formed elements was within normal limits.

**Table 2:** Blood and urinalysis parameters of the patient.

**Test Type**	**Parameter**	**Result (2021)**	**Result (2022)**	**Physiological range**
CBC	RBC (millions/μL)	7.06	N/A	5.65–8.87
	HCT (%)	46.6 %	N/A	37.3–61.7
	HGB (g/dL)	15.6	N/A	13.1–20.5
	WBC ( × 10^3^μL)	10.77	N/A	5.05–16.76
	MONO ( × 10^3^μL)	**1.35**	N/A	0.16–1.12
CMP	Total protein (g/dL)	7.2	**8.3**	5.7–8.0
	Albumin (g/dL)	4.3	4.0	2.8–4.0
	Globulins (g/dL)	2.9	4.3	2.6–4.5
	ALT (GPT) (UI/L)	49	55	15–78
	Alkaline phosphatase (UI/L)	92	81	16–119
	Creatinine (mg/dL)	0.91	0.95	0.5–1.8
	BUN (mg/dL)	14	19	7–23
Urinalysis	Specific gravity	1.015	1.030	1.020–1.050
	pH	6	7	5.5–7.5
	Sediment (casts)	Absent	Moderate	Absent
	Protein	0	0	0–30 mg/dL

Parameters that exceed the normal ranges are in bold. CBC, Complete Blood Count; RBC (millions/L), Red Blood Cells; HCT (%), Hematocrit; HGB (g/dL), Hemoglobin; WBC ( × 10^3^μL), White Blood Cells; MONO ( × 10^3^μL), Monocytes; CMP, Comprehensive Metabolic Panel; ALT (GPT) (UI/L), Alanine Aminotransferase (Glutamate Pyruvate Transaminase); BUN (mg/dL), Blood Urea Nitrogen; N/A, not assessed (test not repeated).

### 3.2 Diagnostic imaging

Radiographic imaging revealed a constellation of findings initially interpreted as suggestive of a multifocal neoplastic process. A focal osseous lesion measuring approximately 2 cm was identified in the diaphysis of the right radius (Supplementary Figure S1). The lesion showed a proliferative pattern involving both cortical and medullary bone. Thoracic radiographs demonstrated a diffuse reticular pattern consistent with non-specific interstitial lung disease, together with a focal area of increased opacity compatible with a pulmonary nodule or mass (Supplementary Figure S2). At this stage, these findings were considered non-specific, and differentials included neoplastic, inflammatory, or infectious processes. Abdominal ultrasonography did not reveal abnormalities that could explain the reported urinary incontinence. Computed tomography (CT) allowed further characterization of the skeletal and soft tissue lesions. The radial lesion displayed aggressive features, including mixed lytic and sclerotic changes, marked periosteal reaction, and surrounding soft tissue edema (Supplementary Figure S3). Based on imaging alone, primary or secondary bone neoplasia was considered the leading differential diagnosis. Additionally, CT identified multiple lytic lesions affecting the thoracic spine, particularly the T8 and T9 vertebrae (Supplementary Figure S4), associated with a paravertebral soft tissue mass and focal bone destruction. These findings raised concern for metastatic disease or a primary spinal neoplasm, while infectious osteomyelitis was also considered among the differential diagnoses. Increased soft tissue density within the left frontal sinus (Supplementary Figure S5) represented a non-specific finding, with differential diagnoses including sinusitis, inflammatory disease, or fungal infection. Moderate pre-scapular and axillary lymphadenopathy was interpreted as potentially reactive; however, in the context of multifocal skeletal lesions, a neoplastic etiology was also considered. Degenerative changes of the vertebral column, including intervertebral disc space narrowing, sclerosis, and vacuum phenomenon, were consistent with age-related alterations.

### 3.3 Cytological and histopathological examination

Fine-needle aspiration cytology and biopsy specimens were obtained directly from the osseous lesion of the right radius using a Jamshidi needle. No soft tissue involvement was observed during imaging or sampling. Cytological evaluation revealed a mixed inflammatory infiltrate composed predominantly of neutrophils and macrophages, with evidence of fungal hyphae. Microscopically there are septate hyaline hyphae that measure 1.5–3 μm in diameter and from which mono- and bi-verticillate conidiophores arise showing flask-shaped phialides with tapered tip, no metulae and oval/rounded conidia in short chains. Histopathological examination of the bone tissue confirmed chronic fibroplasia and fibrosis associated with mixed inflammation. PAS and GMS staining highlighted fungal hyphae within necrotic areas. Both stains revealed the presence of fungal hyphae, characterized by rounded and elongated structures within the necrotic debris (Supplementary Figure S6).

### 3.4 Microbiological investigations

No bacterial growth was observed on routine culture media. However, after 4–5 days of incubation, a pure fungal culture emerged on SABC medium, while no growth was detected on blood agar. The fungal colony exhibited a characteristic white to grayish appearance with a tendency to develop aerial mycelia (Supplementary Figure S7). Colonies showed a velvety texture and were initially flat and whitish, becoming progressively raised and grayish-brown with age. The reverse surface exhibited pale pigmentation, ranging from whitish to light cinnamon with cream-colored shades. Occasional small hyaline droplets were observed on the colony surface. Microscopic examination revealed the presence of septate hyphae and conidiophores consistent with the genus *Penicillium* (Supplementary Figures S8A, B). Subsequent urine cultures, collected at two-month intervals during therapy, consistently yielded fungal isolates with identical morphological characteristics to the original *Penicillium* species, suggesting persistent infection. Molecular identification using PCR amplification and Sanger sequencing of the β-tubulin, calmodulin, and nuclear ITS regions confirmed the persistent presence of the same *Penicillium* species in these cultures.

### 3.5 Molecular identification

The identification of the fungus was confirmed molecularly by PCR amplification and sequencing of β-tubulin (GenBank accession number PV335504), calmodulin (GenBank accession number PV288913), and nuclear ITS region (GenBank accession number PV133825). The same fungus was identified in other biological samples (biopsy and urine samples). Sequencing of β-tubulin, calmodulin, and ITS regions from both urine and bone biopsy samples yielded identical nucleotide sequences across all loci, confirming the persistence of the same *P. labradorum* strain in different tissues. Briefly, all sequences were queried against the GenBank database. The analysis showed 99.48% nucleotide identity in ITS to *Penicillium parvum* strain NRRL 2095 (GenBank accession number AF033460.1). The analysis of the calmodulin gene displays an identity of 97% nucleotide identity to *P. labradoris* strain DI19-20 (GenBank accession number MK887899.1). A nucleotide identity of 100% was detected in the β-tubulin to *Penicillium labradorum* tt14509 (GenBank accession number ON646037.1). Phylogenetic analyses based on three distinct gene targets demonstrated that the fungal strain isolated from the dog consistently clustered with *P. labradorum* tt14509 (GenBank accession number ON646037.1), *P. labradoris* CBS 145775 (GenBank accession number NR173380), and *Penicillium striatisporum* NRRL 26877 (GenBank accession number NR173380) in the internal transcribed spacer (ITS) region (Supplementary Figure S9). Analysis of the β-tubulin gene revealed that the strain grouped closely with *P. labradorum* tt14509, while phylogenetic reconstruction based on the calmodulin gene also supported clustering with *P. labradorum* tt14509 and *P. labradoris* CBS 145775 (Supplementary Figures S10, S11).

## 4 Discussion

This report presents the first documented case of disseminated *P. labradoris* infection in an Italian German Shepherd. The diagnosis required a multidisciplinary approach, and despite therapeutic intervention, the outcome was, unfortunately, not favorable. This case underscores the importance of recognizing and addressing this emerging fungal pathogen in veterinary medicine. Initially, the dog presented with a non-painful, firm swelling on the right forelimb. No significant underlying immunosuppressive conditions or recent drug therapies, such as corticosteroids or immunosuppressants, were identified. Phenylpropanolamine, which was administered for urinary incontinence, is not known to have significant immunosuppressive effects. Therefore, the presence of a fungal infection in this seemingly immunocompetent dog highlights the potential for opportunistic fungal infections in dogs, even without overt immunosuppression. *Penicillium* species are ubiquitous environmental fungi, but only a few species have been recognized as human and animal pathogens (1). Interestingly, *P. canis* and *P. labradorum* (syn. *P. labradoris*) have been implicated in canine osteomyelitis and disseminated infections, respectively (3, 5, 6, 22). In humans, *P. decumbens* has been associated with invasive pulmonary infections, fungemia, and disseminated disease (7). The clinical presentation of canine *Penicillium* infections can vary but often involves osteomyelitis, disseminated disease, pneumonia, and encephalitis (3). In the present case, the non-painful soft tissue swelling was an atypical presentation, emphasizing the need for a high index of suspicion for fungal infections, even in the absence of classic clinical signs. While immunosuppression is a recognized risk factor for fungal infections, recent studies have highlighted the role of genetic predisposition in canine fungal infections (1, 3, 5). German Shepherds, chiefly young females, and Labrador Retrievers appear to be overrepresented among cases of disseminated fungal infections, suggesting a potential genetic susceptibility (3, 11). Genetic factors, such as polymorphisms in immunomodulatory genes (i.e., including *STAT1, STAT3, CD40L, IFNGR1*, and *CARD9*), may influence the host's response to fungal infections (3, 23–25). The portal of entry for *Penicillium* infection in dogs is often unclear, but potential routes include inhalation, cutaneous inoculation, and ingestion (1, 5, 26). In the present case, the exact route of infection remains speculative. The diagnosis of disseminated fungal infections in dogs often relies on a combination of clinical, radiological, and laboratory findings. In this case, radiographic and CT imaging revealed osseous lesions and soft tissue involvement. Histopathological examination of the biopsy tissue demonstrated the presence of fungal hyphae, while fungal culture and molecular identification techniques were essential for definitive species-level identification of the *Penicillium* isolate. In addition to culture from biopsy material, urine culture proved to be a valuable, non-invasive diagnostic tool in the present case. Persistent funguria, documented through repeated urine cultures during antifungal therapy, provided microbiological evidence of ongoing infection and has been reported as a useful diagnostic approach in other cases of canine systemic mycoses. Moreover, given the opportunistic and environmental nature of *Penicillium* species, histopathology played a pivotal role in confirming the fungal etiology by directly demonstrating fungal elements within affected tissues, thereby strengthening the multidisciplinary diagnostic approach adopted. To date, only a limited number of *Penicillium* infections have been reported in dogs. Early descriptions include pulmonary infection caused by *P. commune* (27) and osteomyelitis associated with *P. canis* (5). Later, (6) documented disseminated infection caused by *P. labradorum* in a Labrador Retriever, and three additional cases involving bone lesions were more recently reported by (22) in Western Australia. Notably, all three cases exhibited indolent progression and responded to prolonged antifungal therapy (e.g., posaconazole), though surgical intervention was required in some instances. This clustering of cases in Western Australia raises questions about potential environmental or genetic predisposing factors (22). These studies, together with the present case, expand the known clinical spectrum of *P. labradorum* infection in dogs and support its recognition as an emerging opportunistic pathogen in veterinary medicine. A few unspecified cases of canine penicilliosis have also been described (28), and osteomyelitis appears to be a common manifestation of systemic fungal dissemination. In contrast, while *Aspergillus* spp. infections share some clinical similarities, they are far more common and have been widely documented in dogs with systemic or localized disease (29, 30). The focus of this report, however, remains on *Penicillium* species as uncommon but clinically significant pathogens capable of mimicking other invasive mycoses. The respiratory or gastrointestinal system are proposed as the entry point and systemic infection generally occurs via the dissemination of circulating spores (3, 6). Usually, these infections occur in severely immunocompromised hosts. However, according to a review paper, out of 157 cases of disseminated mold infections in dogs, only 30 (19.1%) dogs had a history of immunosuppressive therapy, and the remaining cases did not report predisposing factors (2). The treatment of disseminated fungal infections is challenging and often unsuccessful. Antifungal therapy is frequently required, typically involving a combination of azoles and amphotericin B. However, the emergence of antifungal drug resistance is a growing concern (1, 9). In this case, the persistent isolation of *P. labradoris* from urine samples, despite antifungal therapy, suggests either potential drug resistance or inadequate treatment.

The main strengths of this case include the multidisciplinary diagnostic approach, combining clinical evaluation, advanced imaging, histopathology, fungal culture, and molecular identification, which allowed reliable species-level confirmation of *P. labradoris*. In particular, sequencing of multiple genetic targets and phylogenetic analysis strengthened the diagnostic accuracy and reduced the risk of misidentification with other invasive molds. In addition, repeated positive urine cultures during antifungal therapy provided clinically relevant evidence of persistent infection and treatment failure. This report also has limitations. As this is a single-case description, the findings cannot be generalized to the wider canine population. Furthermore, a complete necropsy was not performed, which limited confirmation of the full extent of dissemination and precluded definitive assessment of organ involvement. Finally, antifungal susceptibility testing and validated fungal biomarkers (e.g., galactomannan or β-D-glucan assays) were not available at the time of diagnosis and follow-up, restricting therapeutic monitoring.

In conclusion, disseminated mycoses caused by different fungal genera, including *Aspergillus* and *Penicillium*, may present with overlapping clinical and imaging features, making definitive diagnosis challenging. For this reason, fungal culture and species-level identification remain essential to confirm the etiological agent and guide appropriate clinical interpretation. Optimizing diagnostic algorithms and expanding epidemiological data will be crucial to better define the pathogenic role of uncommon opportunistic fungi in dogs and to improve strategies for early recognition and management of systemic fungal infections.

*Penicillium* species should be regarded as emerging opportunistic pathogens in dogs, and fungal culture combined with molecular identification should always be considered in cases of unexplained osteomyelitis or suspected disseminated fungal disease.

## Data Availability

The datasets presented in this study can be found in online repositories. The names of the repository/repositories and accession number(s) can be found in the article/Supplementary material.
