# Supplementary material for: Diagnostic odyssey of a German shepherd dog with disseminated Penicillium labradoris infection: a case report
Source: Front Vet Sci. 2026 Mar 6;13:1611862. doi: 10.3389/fvets.2026.1611862 (PMC13002354; doi:10.3389/fvets.2026.1611862)
Supplement: Supplementary file 1 [file Supplementary_file_1.pdf]

Supplementary Material

1 SUPPLEMENTARY FIGURES

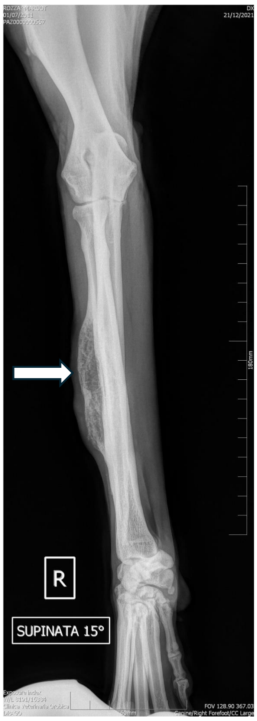

Figure 1a. Mediolateral view (15° supination)

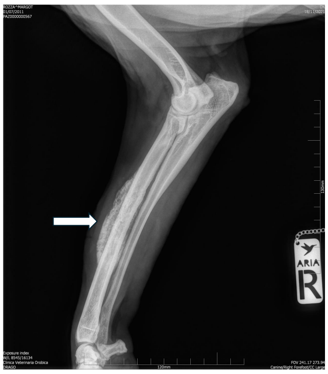

Figure 1b. Craniocaudal view

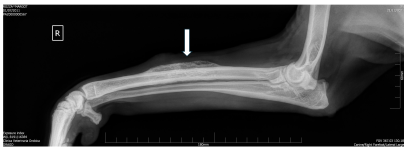

Figure 1c. Lateromedial view

Figure 1. X-ray examination of the right radio revealed a 2 cm diaphyseal proliferative bone lesion (arrow) that also affects the cortical bone. **(A)** Mediolateral projection of the right antebrachium in 15° supination: demonstrates well-aligned radius and ulna with multifocal, irregular periosteal new bone formation and cortical disruption, indicative of an aggressive bone lesion. **(B)** Craniocaudal projection of the right antebrachium: shows extensive periosteal proliferation and heterogeneous intramedullary opacity suggestive of a mixed productive and lytic bone process.**(C)**Lateromedial projection of the right elbow and antebrachium: reveals periosteal and endosteal reactive changes affecting the distal humerus, proximal radius, and ulna, while the elbow joint space remains preserved.

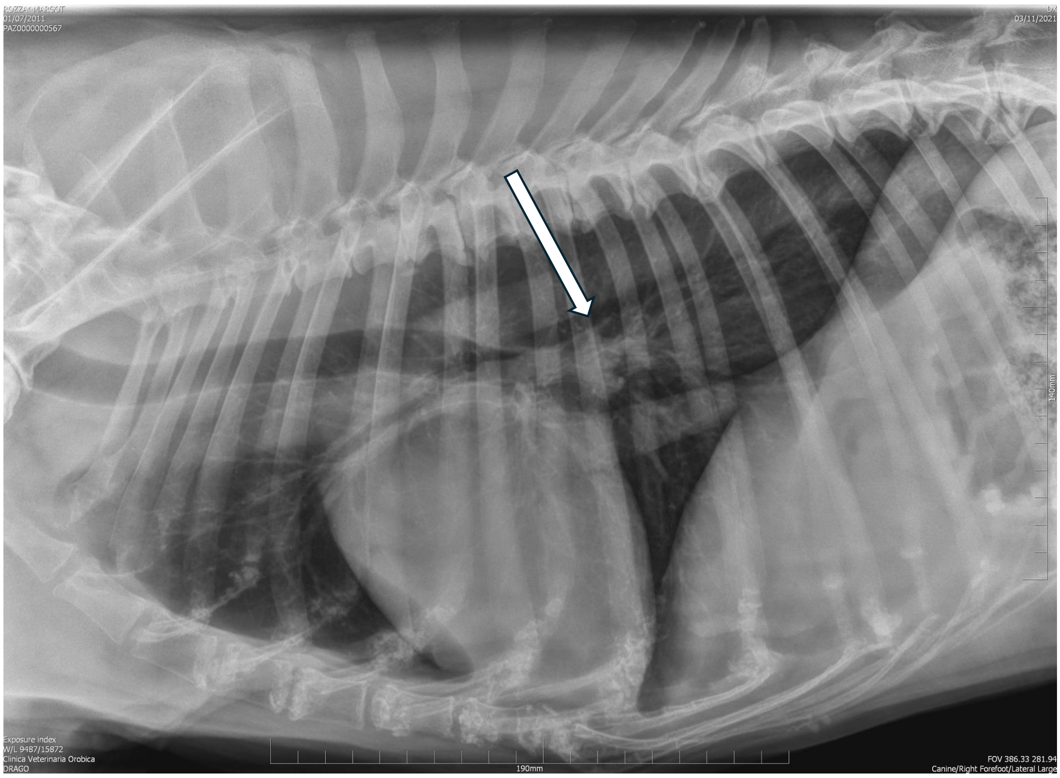

Figure S2. Lateral thoracic X-ray examination.

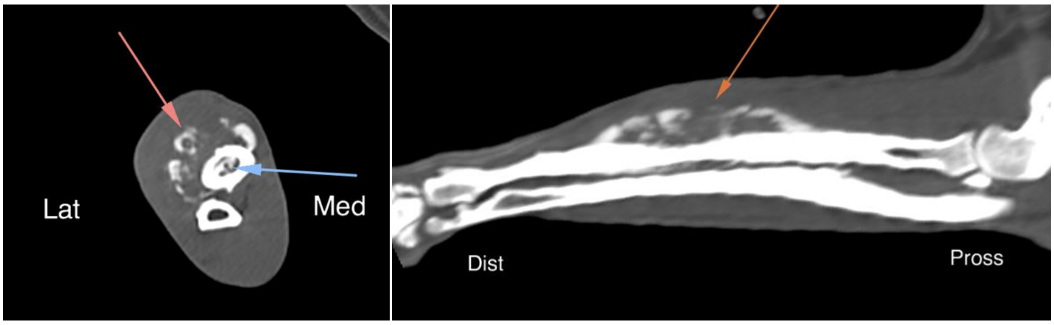

Figure S3. Computerized tomography: Transverse plane image at the level of the middle third of the radial shaft (left) and MPR reconstruction along the long axis of the right radius (right) with hard tissue window. The red arrows indicate the irregular and interrupted bony proliferations dorsal to the middle third of the diaphysis of the right radius. The blue arrow indicates an area with increased density at the level of the medulla (Lat=lateral, Med=medial, Pross=proximal, Dist=distal).

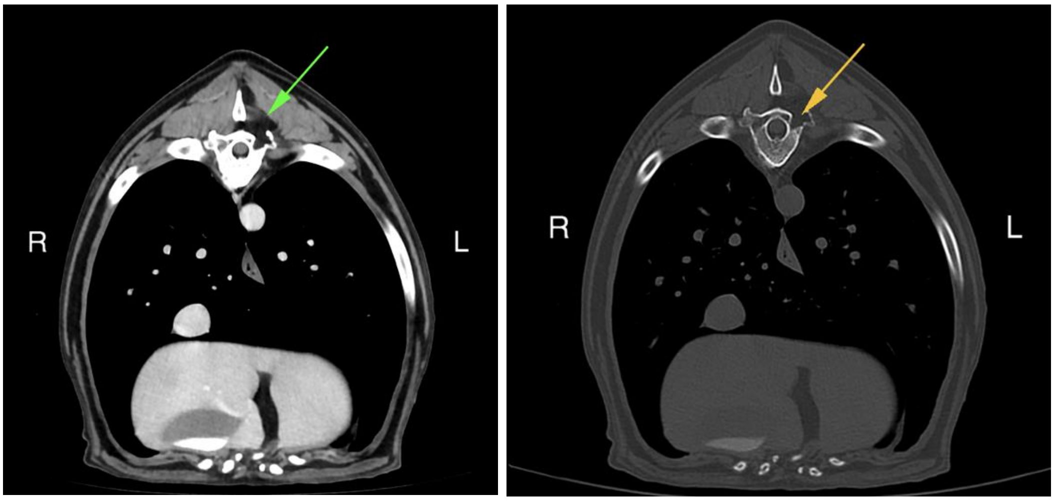

Figure S4. Computerized tomography: Images in the transverse plane at the level of the caudal third of the thorax with a soft tissue window in the post-contrast phase (left) and with a hard tissue window (right). The green arrow indicates the left paravertebral neoformation with predominantly adipose density, the yellow arrow indicates the area of bone lysis/atrophy affecting T9 (R=right, L=left).

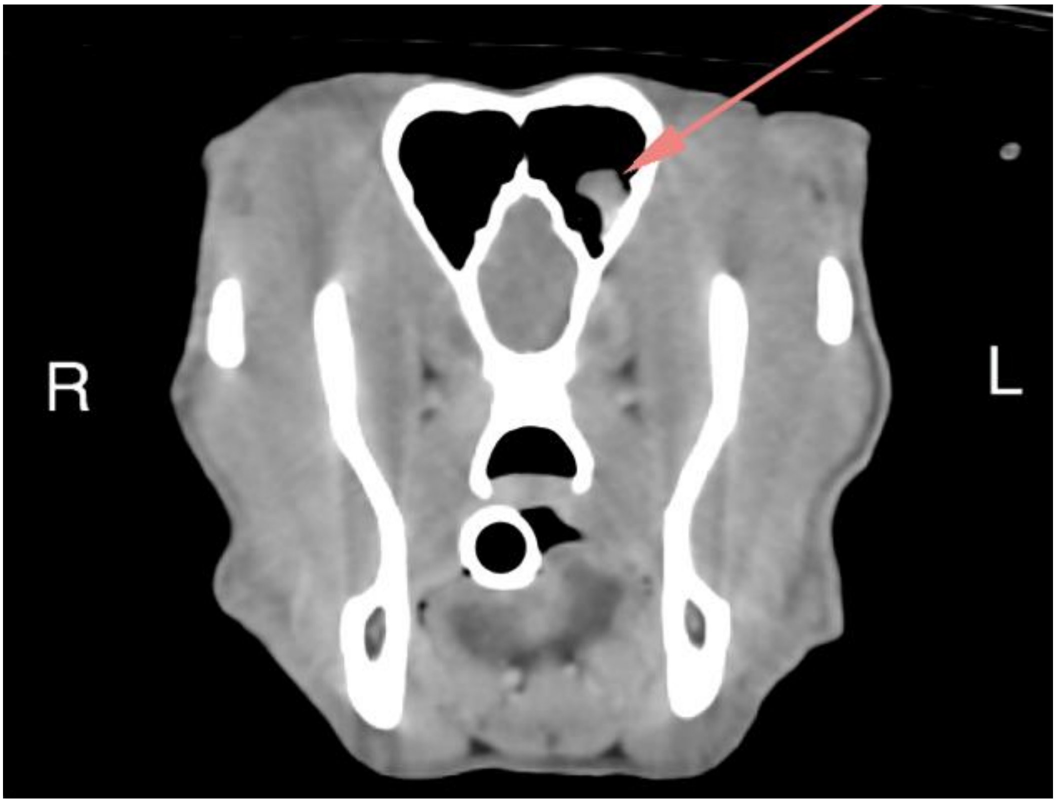

Figure S5. Computerized tomography: Transverse plane image with soft tissue window at the level of the frontal sinuses. The arrow indicates the area with soft tissue density in the left frontal sinus (R=right, L=left).

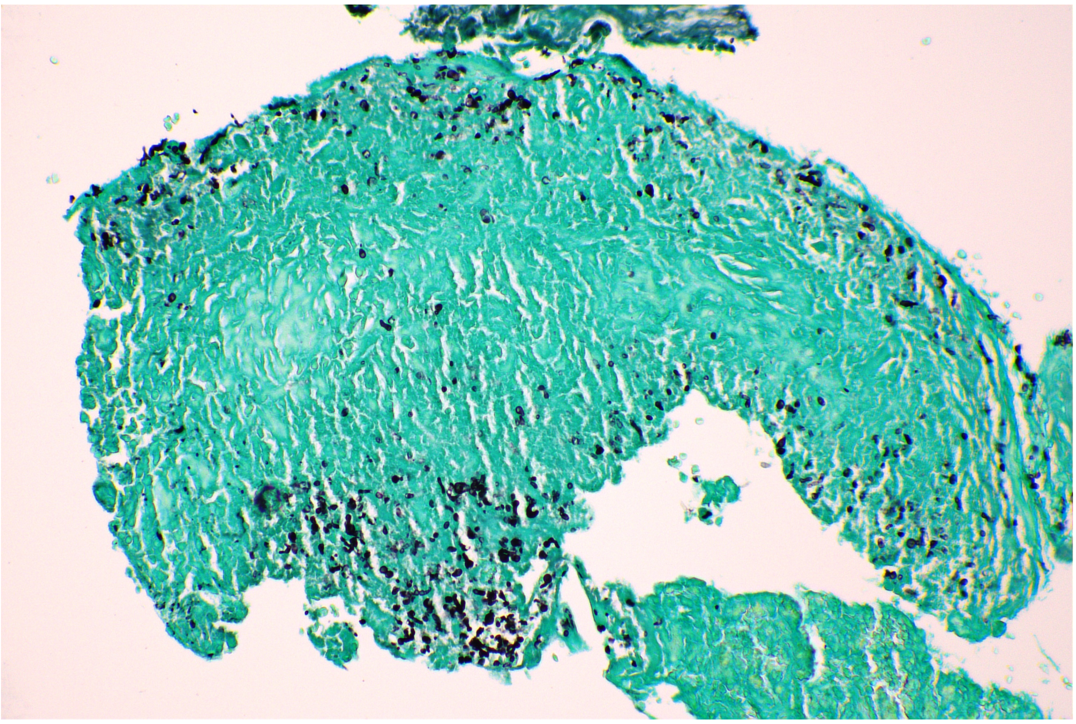

Figure 6a.

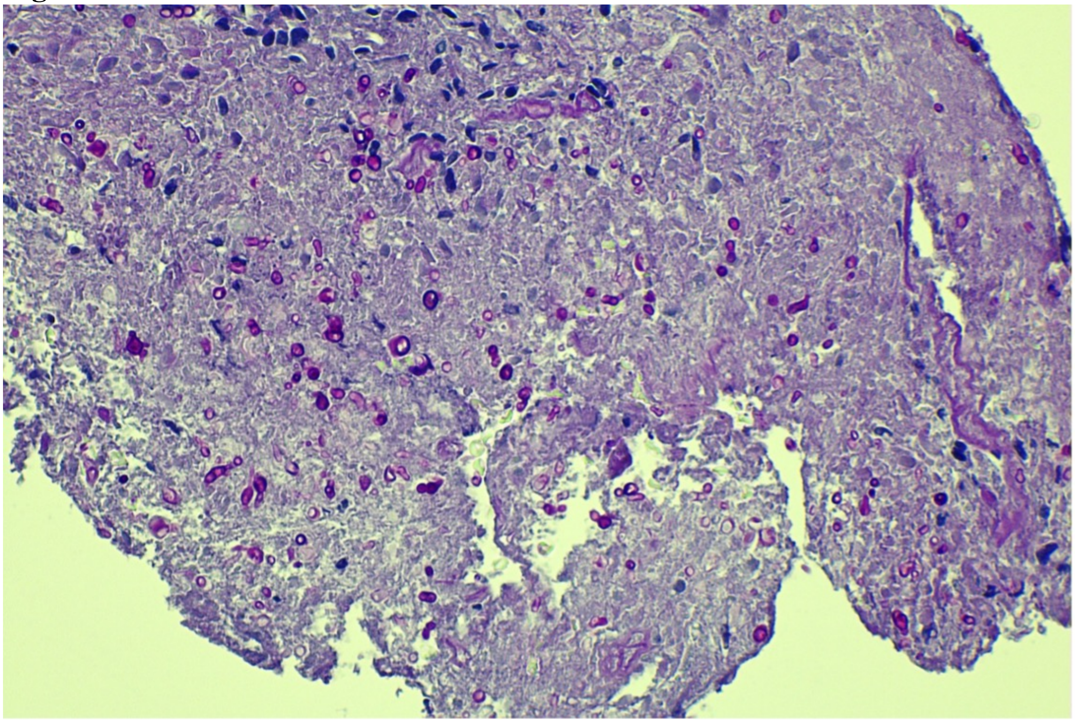

Figure 6b.

Figure 6. **(A)** Grocott staining, bone lesion biopsy, 10X magnification **(B)** PAS staining, bone lesion biopsy, 20X magnification

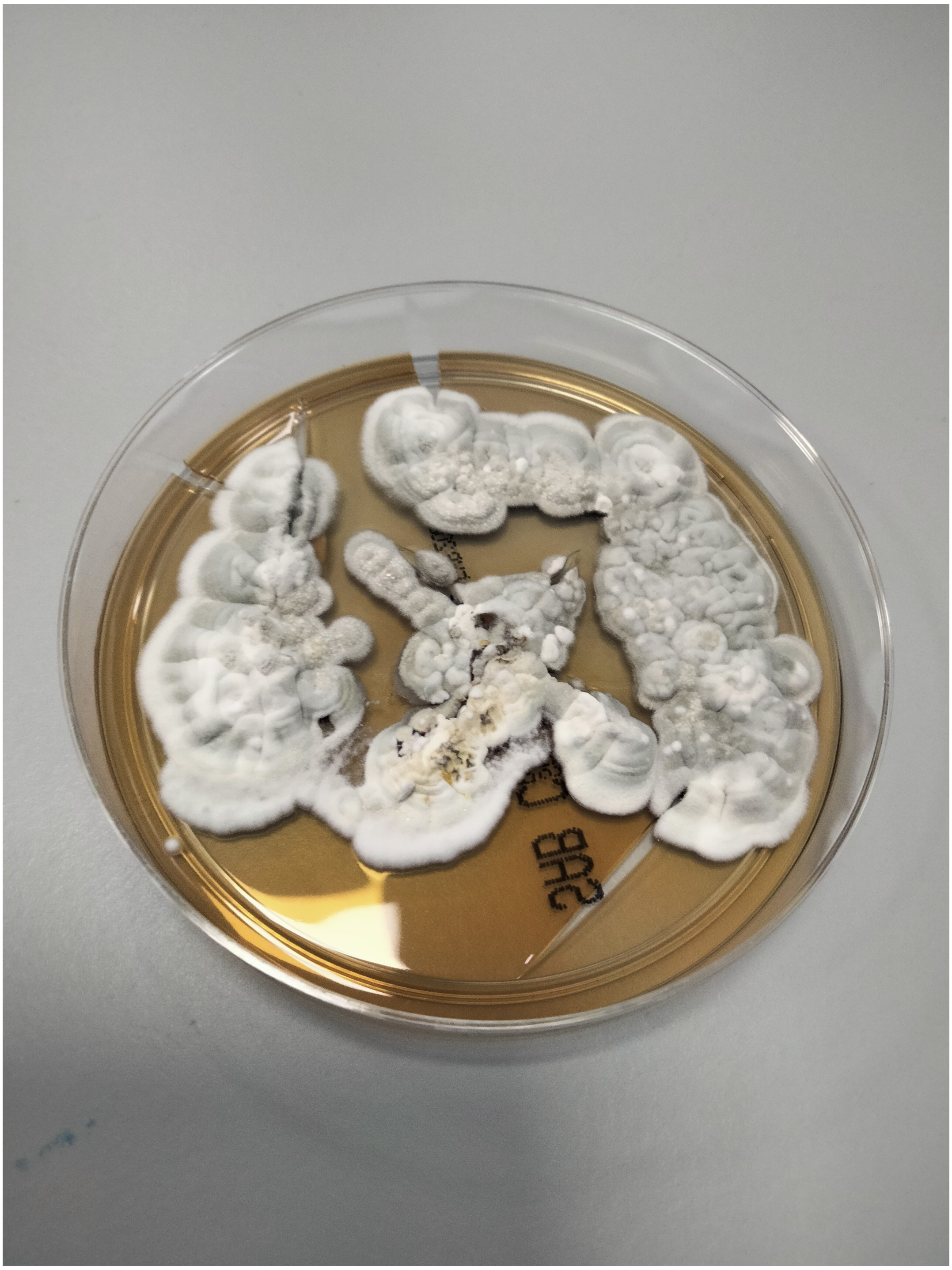

Figure S7. *Penicillium labradorum* on Sabouraud Glucose Agar with Chloramphenicol after 10 days of incubation at 37°.

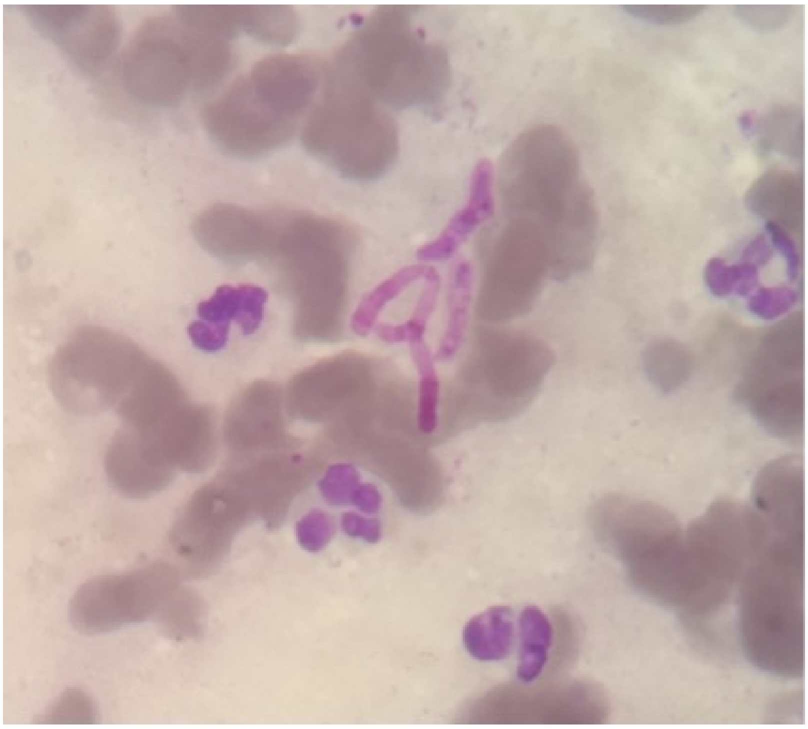

Figure 8a.

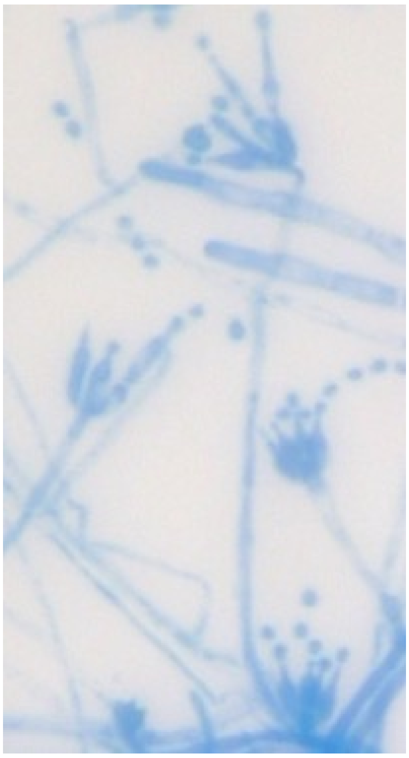

Figure 8b.

Figure 8. **(A)** Bone lesion aspiration cytology, May-Grunwald Giemsa, 100X magnification**(B)** Lactophenol Cotton Blue Staining, fresh colonies from SABC, 100X magnification

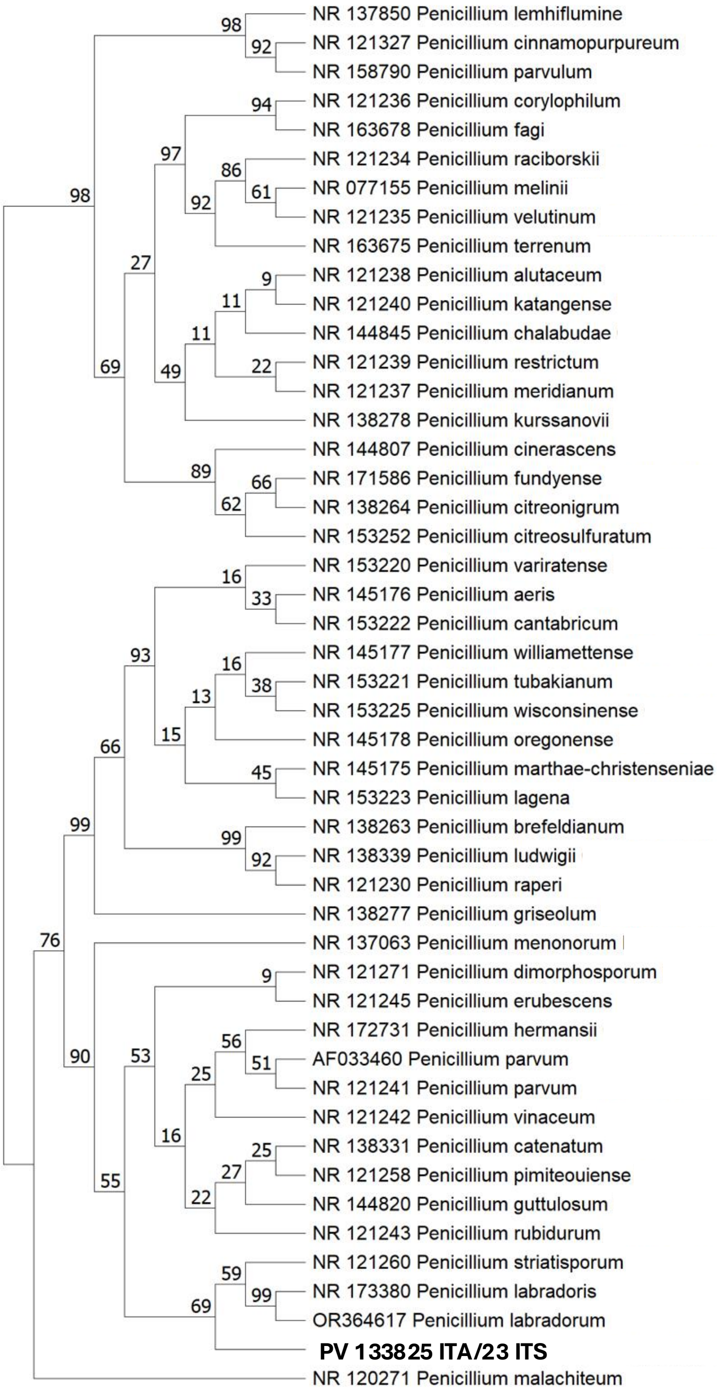

Figure S9. Phylogenetic tree based on Internal Transcribed Spacer (ITS) gene (partial-length), of different *Penicillium* species retrieved from the GenBank database. GenBank accession numbers are provided for reference strains. The tree was generated using the maximum likelihood method, Tamura-Nei model with a gamma distribution, and bootstrapping up to 1000 replicates. Bootstrap support values, representing the percentage of trees in which the associated taxa clustered together, are indicated next to the branches. The percentage of trees in which the associated taxa clustered together is shown next to the branches. Evolutionary analyses were conducted in MEGA11. The strain identified in this study is in bold.

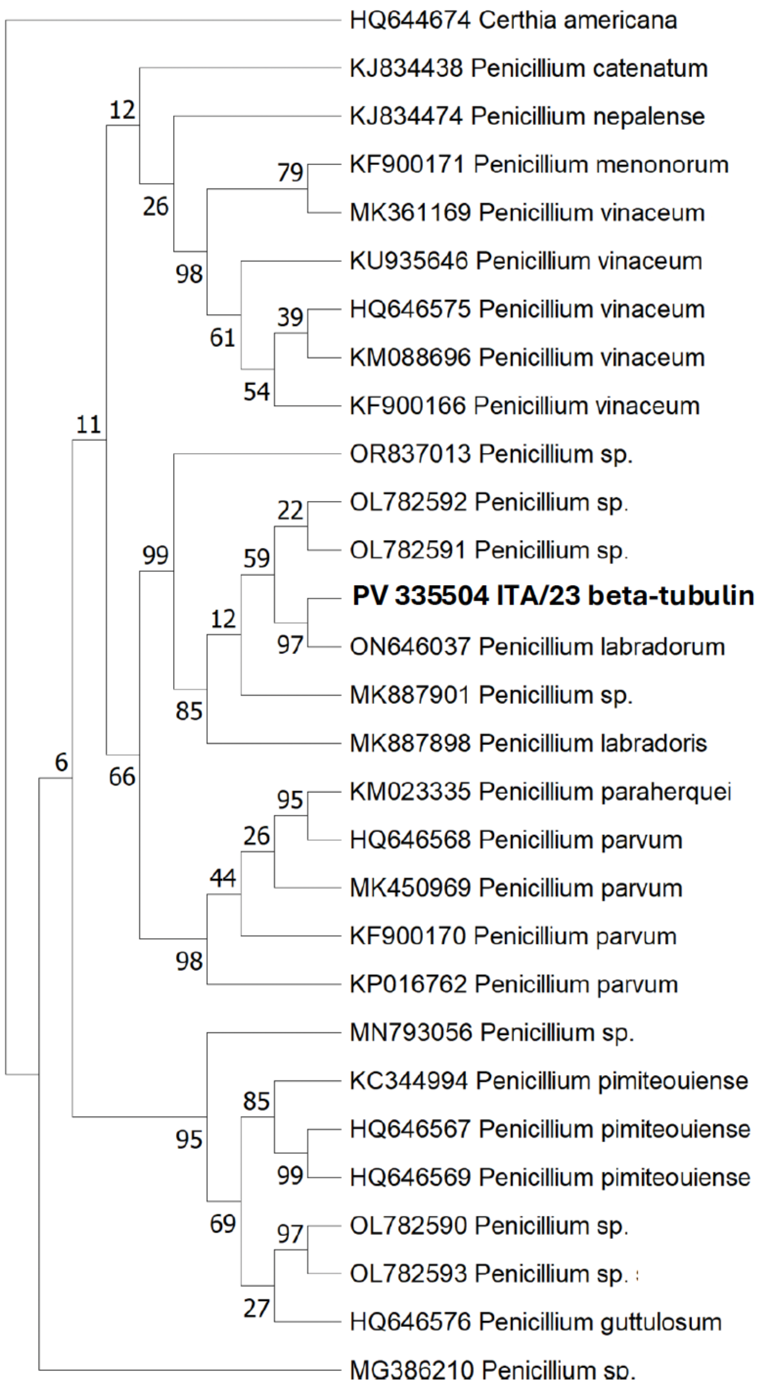

Figure S10. Phylogenetic tree based on Beta-tubulin gene (partial-length), of different Penicillium species retrieved from the GenBank database. GenBank accession numbers are provided for reference strains. The tree was generated using the maximum likelihood method, Hasegawa-Kishino-Yano model with a discrete gamma distribution, and bootstrapping up to 1000 replicates. Bootstrap support values, representing the percentage of trees in which the associated taxa clustered together, are indicated next to the branches. The percentage of trees in which the associated taxa clustered together is shown next to the branches. Evolutionary analyses were conducted in MEGA11. The strain identified in this study is in bold.

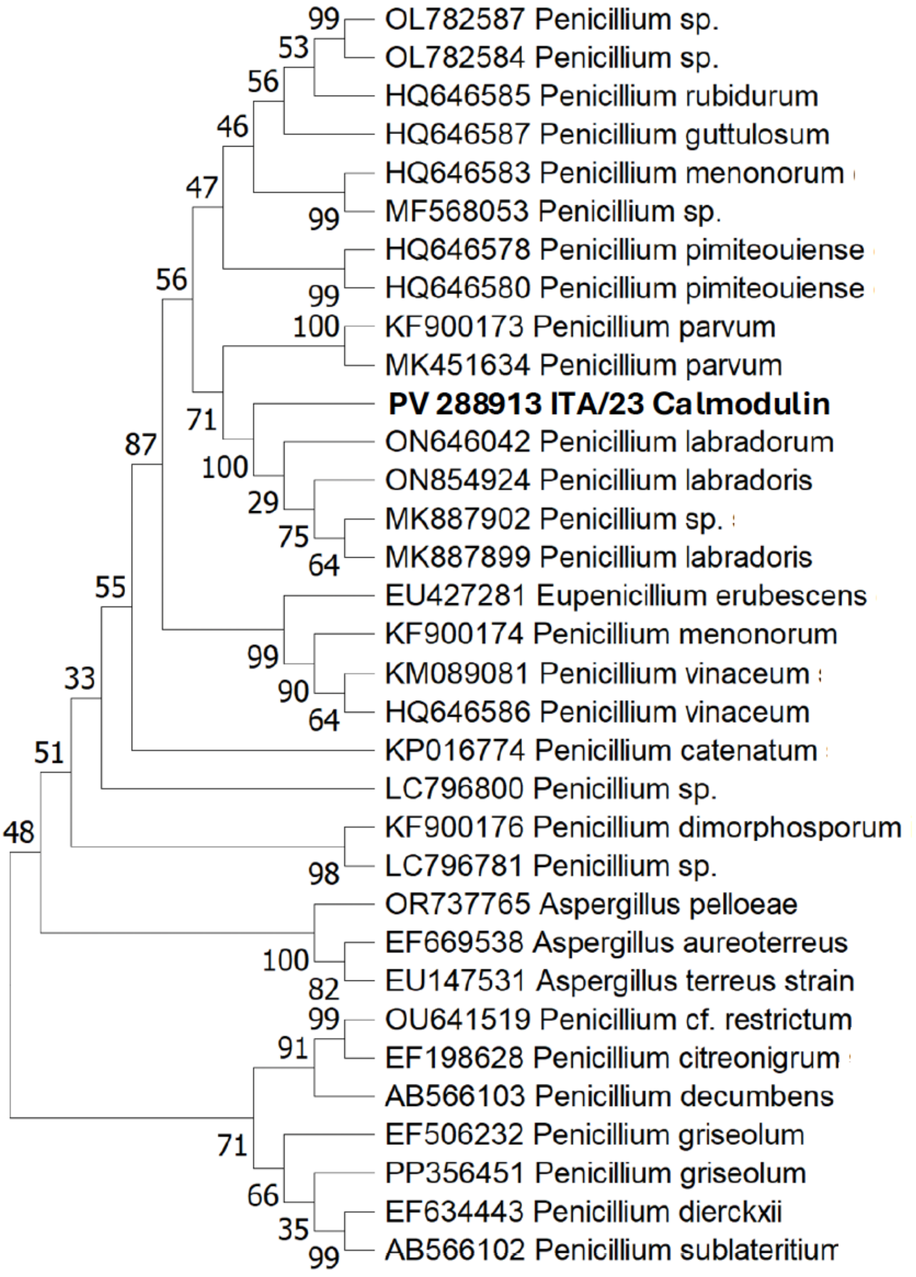

Figure S11. Phylogenetic tree based on Calmodulin gene (partial-length), of different Penicillium species retrieved from the GenBank database. GenBank accession numbers are provided for reference strains. The tree was generated using the maximum likelihood method, Tamura-Nei model with a gamma distribution, and bootstrapping up to 1000 replicates. Bootstrap support values, representing the percentage of trees in which the associated taxa clustered together, are indicated next to the branches. The percentage of trees in which the associated taxa clustered together is shown next to the branches. Evolutionary analyses were conducted in MEGA11. The strain identified in this study is in bold.
